# Synthesis, Quality Control and Preliminary Activity Evaluation of a New Compound HM475

**DOI:** 10.3390/molecules28093753

**Published:** 2023-04-27

**Authors:** Jieqing Guo, Luming Xie, Jing Zhang, Han Cao, Juanxia Wang, Xia Wu, Yifan Feng

**Affiliations:** 1 New Drug Research and Development Center, Guangdong Pharmaceutical University, Guangzhou 510006, China; 2 Guangdong Provincial Key Laboratory of Advanced Drug Delivery, Guangdong Provincial Engineering Center of Topical Precise Drug Delivery System, Guangdong Pharmaceutical University, Guangzhou 510006, China

**Keywords:** honokiol derivatives, metformin, quality control, activity evaluation, neurodegenerative diseases

## Abstract

Based on the principle of molecular splicing and theory of traditional Chinese medicine pairs, a new multi-active compound (HM475) was synthesized by connecting metformin with honokiol, and its structure was characterized, which not only reduced the toxicity of raw materials, but also maintained the original activity, and had a certain significance in research and innovation. At the same time, quality control and preliminary activity evaluation were carried out, and the effect of HM475 on neuroinflammation was further explored, which provided a new idea for drug development of neurodegenerative diseases.

## 1. Introduction

**Figure 1:**
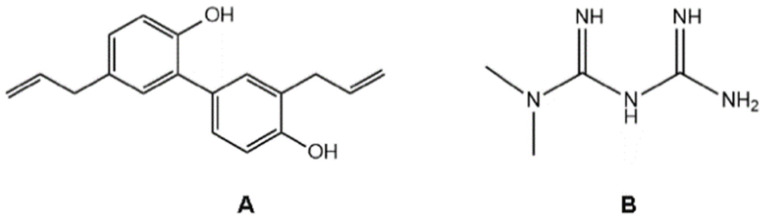
Structure of Honokiol (**A**) and Metformin (**B**).

Honokiol is a natural product that contains allylbiphenol isolated from Magnolia officinalis, which is isomeric with magnolol and is one of the effective components of traditional Chinese medicine Magnolia officinalis. It has a wide range of biological activities [1]; studies have found that Honokiol has anti-tumor [2], anti-oxidation [3], neuroprotective [4], anti-inflammatory [5] and anti-arrhythmic properties [6]. The chemical structure is shown in Figure 1A. In recent years, honokiol has shown good biological activity in neurodegenerative diseases [7,8,9]. It has been reported that honokiol can inhibit the activation of the NF-κB pathway in mouse B cells [10], macrophages [11] and LPS-stimulated glial cells. Furthermore, it can alleviate LPS-induced neuroinflammatory response and reduce the expression levels of related pro-inflammatory factors, which in turn affect tryptophan metabolism and increase neuroprotective metabolites [12,13]. PC12 cells (rat adrenal medullary chromaffin tumor cell line differentiation), have the general characteristics of neuroendocrine cells and are widely used in neurophysiology and neuropharmacology [14]. Meanwhile, the effect of MPTP (1-methyl-4-phenyl-1,2,3,6-tetrahydropyridine) is similar to that of human Parkinson’s disease in many aspects, such as behavior, biochemical pathology, and so on. Therefore, MPTP is widely used as an inducer for animal and cell models of Parkinson’s disease [15]. BV-2 cells and PC-12 cells are commonly used in neurodegenerative disease research such as Parkinson’s disease (PD) [16].

However, Honokiol also has some disadvantages such as high toxicity, low water solubility, poor absorption in vivo, low bioavailability and unstable properties, which greatly limits its clinical use [17]. Since the initially extracted metformin has the advantages of anti-diabetic and small side effects, it has been used for clinical treatment of type 2 diabetes mellitus (T2DM) as early as the 1950s. Metformin has also been found to be effective in treating cardiovascular disease and malignancies. Its chemical structure is shown in Figure 1B. However, metformin has the disadvantages of a short half-life and rapid metabolism; long-term use of metformin may stimulate the gastrointestinal tract, causing abdominal pain, severe diarrhea and vomiting [18]. Taking metformin also prevents the absorption of vitamin B12 [19]. These deficiencies limit the clinical use of metformin. Therefore, structural modifications of honokiol and metformin are of great significance [20].

The theory of traditional Chinese medicine pairs is based on the performance and efficacy of the two drugs, and is selectively combined and compatible, so that the components of the drugs can achieve synergistic synergy, compatibility and detoxification, or new therapeutic effects through interaction [21,22]. The theory of traditional Chinese medicine is a summary of the long-term clinical experience of physicians in the past dynasties [23]. Guided by the theory, we propose the design of multinuclear molecules. At present, most of the structural modifications of active ingredients or compounds of traditional Chinese medicines are mainly to introduce hydrophilic or lipophilic small molecular fragments into their original structures [24,25,26]. The multi-nuclear molecule design is to connect the active “natural mononuclear molecules” in traditional Chinese medicine to form multi-nuclear molecules with multiple core functions by artificial splicing to enhance the efficacy of drugs, increasing the drug function target and symptomatic treatment [27,28]. This multi-nuclear molecule not only has the characteristics of a single “natural mononuclear molecule”, but also has the characteristics of multiple “natural mononuclear molecules”, so that the new compound formed can remain its biological activity and improve the shortcomings of the original ingredient, even increasing its activity [29,30]. This method provides a new idea for the synthesis and activity study of new compounds.

Due to the significant activities of honokiol and metformin in disease research and the toxic and side effects of both, this topic is based on the research idea of multinuclear molecular design. Making honokiol which is the active ingredient isolated from the traditional Chinese medicine Magnolia officinalis, and metformin chemical splicing. Then, a new compound HM475 was obtained and the structure of new compound was determined by UV-Vis, IR, HRMS and NMR, and its relative structure was determined by X-ray single crystal diffraction. Under the conditions of strictly following the Chinese Pharmacopoeia (2020) [31], we have carried out quality control on HM475, including the determination of physical and chemical properties, impurity inspection, content determination, and influencing factor experiments. At the same time, the anti-inflammatory, anti-insulin resistance (IR), neuroprotective and anti-breast cancer activities of HM475 in vitro were evaluated, which had important research significance and innovation in the research and development of new drugs.

## 2. Results and Discussion

### 2.1. Synthesis of ***3a***

**Scheme 1:**
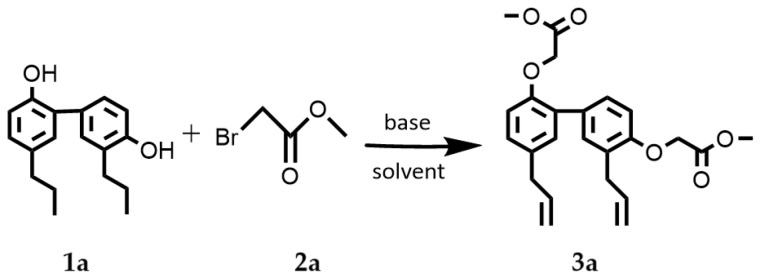
Synthetic route of **3a**.

The conditions of reaction solvent, base, and reaction temperature were optimized. During the synthesis process of compound **3a**, the carbonyl group was connected to honokiol through bromoalkylation (Scheme 1). methanol, ethanol, isopropyl alcohol (IPA) and acetonitrile were used as solvents to investigate the effects of different solvents on the yield. The result in the Table 1 showed that the reaction was easier in protic solvents and the yields were higher (entries 1–3). To make the reaction react completely, an acid-binding agent was selected to be added, and the acid-binding agent includes sodium carbonate and potassium carbonate. The yield was highest when using sodium carbonate (entries 4–11). Therefore, the optimal reaction conditions were found to involve methanol as a solvent with 2.0 equivalents of Na_2_CO_3_ as base at 65 °C. The best yield of **3a** (77.5%) was obtained at these conditions [30]. This experiment provided a new method for honokiol to introduce a carbonyl group.

### 2.2. Synthesis of Compound ***3*** (HM475)

**Scheme 2:**
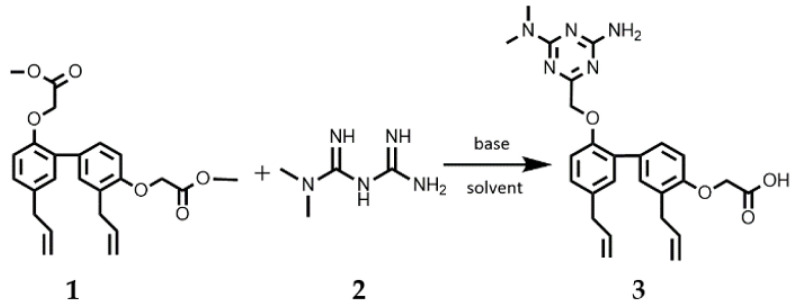
Synthetic route of 3 (HM475).

With the optimized reaction conditions in hand, further optimizations of the reaction conditions for honokiol derivatives (1) and metformin (2) were performed. This reaction was a cyclization reaction that can be carried out in both protic and aprotic solvents, the same as previous nucleophilic reactions; when a protic solvent was selected as the medium, its yield was far superior than aprotic solvent (entries 1–3). Table 2 showed that methanol, as the reaction solvent, exhibited the highest yield (17.8%). The esterified honokiol (compound **1a**) cannot be directly cyclized with metformin in anhydrous methanol solution, so a base catalyst was selected to hydrolyze compound **1** to form a free carbonyl structure to react with metformin (entries 1–13). When the reaction temperature was 65 °C, the reaction yield reached a maximum of 17.8% (entries 14–16). In the Scheme 2, metformin underwent cyclization reaction to form 1,3,5-triazine ring structure derivative compound **3** (HM475).

### 2.3. Characterization of Compound HM475

**Figure 2:**
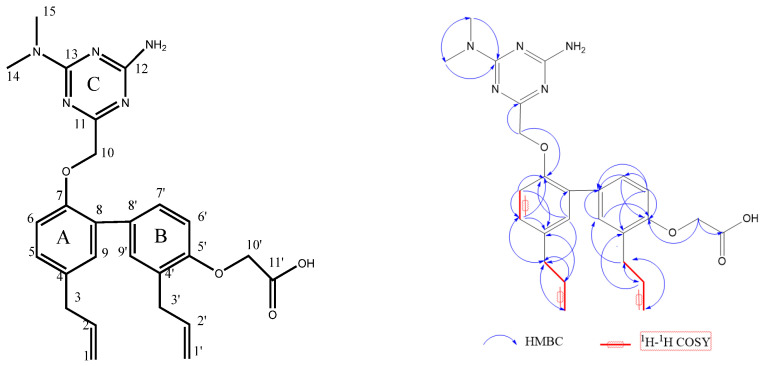
The number of carbon atoms and A, B, C ring (**left**), Key heteronuclear multiple bond correlation (HMBC) and ^1^H-^1^H COSY correlations (**right**).

The chemical structure of 2-((3,5′-diallyl-2′-((4-amino-6-(dimethylamino)-1,3,5-triazin-2-yl)methoxy)-[1,1′-biphenyl]-4-yl)oxy)acetic acid (HM475, C_26_H_29_N_5_O_4_) was characterized by UV-Vis, IR, HRMS, NMR and X-ray single crystal diffraction. In the HRMS spectra, the determined molecular weight is consistent with the calculated value (calculated for C_26_H_29_N_5_O_4_ [M + H]^+^ *m*/*z* = 476.2298, found: *m*/*z* = 476.2294). In the UV-Vis spectra, the maximum absorption peaks at 210 nm and 266 nm were observed (Figure S1, ESI). Infrared spectroscopy (IR) showed that the structure of the compound HM475 has a hydroxyl (3075.9 cm^−1^), hydrocarbon chain double bond (1643.05 cm^−1^), carbonyl (C = O) (1680.66 cm^−1^) and benzene ring (1496.4 cm^−1^, 1578.45 cm^−1^) (Figure S2, ESI). ^1^H-NMR (500 MHz, DMSO-*d*_6_, Figure S3, ESI): δ 3.07 (6H, s, H-14, H-15), 3.31 (2H, d, *J* = 6.5 Hz, H-3′), 3.38 (2H, d, *J* = 7.0 Hz, H-3), 4.72 (2H, s, H-10′), 4.76 (2H, s, H-10), 4.97–5.06 (4H, m, H-1, H-1′), 5.93–5.99 (2H, m, H-2, H-2′), 6.91 (2H, d, *J* = 8.5 Hz, H-6, H-6′), 7.02 (1H, s, H-7′), 7.38 (1H, s, H-9) 7.49 (1H, s, H-9′), 7.06 (1H, s, H-5). ^13^C-NMR (125 MHz, DMSO-*d*_6_, Figure S3, ESI): 172.63 (C-11), 165.08 (C-11′), 166.55 (C-13), 170.32 (C-12), 153.54 (C-7), 127.40(C-7′), 138.05 (C-2′), 136.89 (C-2), 132.96 (C-4, C-4′), 131.86 (C-9), 130.84 (C-9′), 128.24 (C-5), 154.40 (C-5′), 128.24 (C-8, C-8′), 112.12 (C-6),113.86 (C-6′), 115.48 (C-1,C-1′), 70.45 (C-10), 65.87 (C-10′), 38.64 (C-3), 35.01 (C-3′), 35.64 (C-14, C-15). According to ^1^H-^1^H COSY (Figure S4, ESI) and HMBC (Figure S5, ESI), H-1 and H-2 of HM475 were coupled, and H-1 and C-3 were coupled, indicating that C-1 was connected to C-2, and C-2 was connected to C-3. Similarly, H-1′ coupling with H-2′, H-1′ coupling with C-3′, which indicated C-1′ was connected with C-2′ and C-2′ was connected with C-3′. In the Figure 2, COSY and HMBC H-5 of HM475 was coupled with H-6, H-5 was coupled with C-4 and C-7, H-6 was coupled with C-7 and C-5, H-9 is coupled with C-8 and C-7, which can confirm the structure of the A ring. Similarly, H-5′ coupling with C-6′ and C-7′, H-6′ coupling with C-7′ and C-8′, H-9′ is coupled with C-5′ and C-7′, so the structure of B ring can be confirmed. According to H-10 coupling with C-7 and C-11, H-14 coupling with C-13 and C-15, H-15 coupling with C-13 and C-14, the structure of triazine ring C ring can be determined. According to the single crystal diffraction results, the molecular formula of HM475 is C_26_H_29_N_5_O_4_, the ortep diagram is depicted in Figure S10, the crystal data are listed in Table 3, and the selected bond lengths and angles of HM475 can be obtained free of charge from the Cambridge Crystallographic Data Centre (CCDC 2248966).

### 2.4. Studies of Quality Control

We further studied its quality control, on the basis of strictly following the Chinese Pharmacopoeia (2020); its physical and chemical properties, content and impurity were determined. The results showed that HM475 was a lightly yellow powder, odorless and tasteless, insoluble in other common solvents except sodium hydroxide solution. The limit of chloride and iron salt was not less than 0.002% and 0.001%, respectively, in the inspection of impurities. When dried at 105 °C to constant weight, the weight loss did not exceed 0.3%, the residue on ignition did not exceed 0.14%, the limit of heavy metals is less than 10 ppm, the limit of arsenic salt was 1 ppm, and the content determination results showed that the content of HM475 was all above 99.19%. Furthermore, it was stable for 10 days under the conditions of high temperature, high humidity and strong light irradiation, including in accelerated and long-term experiments (Tables S2–S19, ESI).

### 2.5. Evaluation of Anti-Inflammatory Activity of HM475 In Vitro

**Figure 3:**
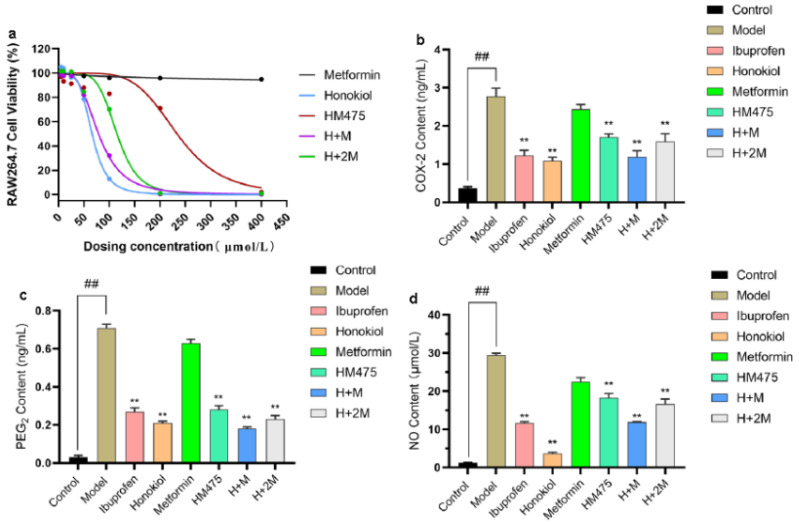
The assays of cell viability of RAW264.7 (**a**), COX-2 content (**b**), pEG2 content (**c**) and NO content (**d**). ## *p* < 0.0001 and ** *p* < 0.01 compared with model group. H + M group was a 1:1 mixture of honokiol and metformin. H + 2M group was administered in a 1:2 mixture.

We also studied its biological activity. Honokiol itself possesses good anti-inflammatory activity, but its application was limited due to its high toxicity. To verify whether the anti-inflammatory activity of honokiol would be lost after structural modification, we chose RAW264.7 cells as the experimental carrier and measured the RAW264.7 cells under the stimulation of lipopolysaccharide (LPS). Under the different drug groups to administrate, whether the concentration of NO, COX and PGE2 changed determined the anti-inflammatory activity of the derivative, and preliminarily evaluated its anti-inflammatory mechanism. There was a significant difference between the model group (LPS) and the blank group, *p* < 0.01, indicating that the model has been successfully established in the experiment. Figure 3a showed that HM475 compared with honokiol, the toxicity was greatly reduced, suggesting that HM475 was less toxic toward RAW264.7 cells. The survival rate data was imported into SPSS software, and the IC_50_ value of the administration group was obtained by Probit analysis. The IC_50_ value of HM475 is 240.43 ± 3.2 μmoL/L, which is only lower than that of the metformin group (IC_50_ > 1600 μmoL/L) and far exceeding the honokiol group (IC_50_ = 66.15 ± 1.74 μmoL/L). RAW264.7 cell survival curves illustrate significantly reduced toxicity of HM475. Subsequent experiments were carried out by selecting drug concentrations with cell viability between 80% and 90% after administration in each administration group. Therefore, Metformin, honokiol and HM475 were administered at 50 μmoL/L. The positive control (Ibuprofen) COX-2 content was 1.23 ng/mL, which was much lower than that of the model group (2.78 ng/mL). The COX-2 content of HM475 (1.71 ng/mL) was slightly higher than ibuprofen group (Figure 3b). The high expression of COX-2 will produce a large amount of the inflammatory factor PGE2. Therefore, we measured the prostaglandin E2 (PGE2) level in inflammatory tissue, and ibuprofen was still used as a positive control drug. The results show that the inhibitory effect of each drug group on PGE2 is similar to that of COX-2, except for the metformin group (Figure 3c), the PGE2 content of 0.28 ng/mL in the HM475 group is slightly higher than in the ibuprofen group (0.27 ng/mL) and mixed administration groups (H + M: 0.18 ng/mL, H + 2M: 0.23 ng/mL). As given in Figure 3d, the NO content in the ibuprofen group was 11.72 μmoL/L, which is lower than that in the model group (29.36 μmoL /L). Hence, HM475 possesses anti-inflammatory activity, indicating that HM475 can inhibit the iNOS enzyme and COX-2 enzyme activity, further reducing the release of pro-inflammatory factors, and producing an anti-inflammatory effect.

### 2.6. Study on the Activity of HM475 on PC-12 and BV-2 Cells

**Figure 4:**
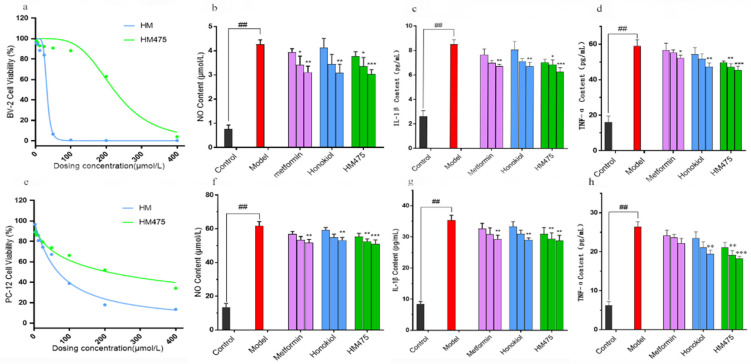
Effect of administration group on cell viability and inflammatory factors of BV-2 cells (**a**) cell viability, (**b**) NO content, (**c**) IL-1β content, (**d**) TNF-α content and PC-12 cells (**e**) cell viability, (**f**) NO content, (**g**) IL-1β content, (**h**) TNF-α content. ## *p* < 0.0001, * *p* < 0.05, ** *p* < 0.01 and *** *p* < 0.001 compared with model group. Three concentrations of HM and HM475 are 3.13 μmoL/L, 6.25 μmoL/L and 12.5 μmoL/L from left to right.

HM475 has excellent anti-inflammatory activity, which prompts us to study the neuroprotective effect of HM475 in neurodegenerative diseases. Taking LPS-activated microglia BV-2 and MPTP (1-methyl-4-phenyl-1,2,3,6-tetrahydropyridine)-activated PC-12 cells as the research objects, the cells were screened by HM475 using the 3-(4,5-dimethylthiazol-2-yl)-2,5-diphenyltetrazolium bromide (MTT) method. Three different concentrations of 3.13, 6.25 and 12.5 μmoL/L (cell survival rate > 80%) were used to treat the above cells for 24 h. The effects of HM475 on NO, TNF-α and IL-1β were detected. Figure 4a,e showed that the cytotoxicity of HM475 to BV-2 and PC-12 cells was significantly reduced compared with the bulk drug honokiol. The IC_50_ value of honokiol corresponding to the two cells was 32.66 ± 2.17 μmoL/L (BV-2) and 76.44 ± 4.55 μmoL/L (PC-12), while the IC_50_ values of HM475 were 221.5 ± 15.67 μmoL/L (BV-2) and 208.4 ± 30.66 μmoL/L (PC-12), respectively. We found that metformin significantly inhibited nitric oxide production but only slightly inhibited tumor TNF-α production, and its anti-inflammatory activity was similar to that of honokiol. However, Figure 4b–h show that the novel conjugates(HM475) has inhibitory effect on inflammatory factors in a concentration-dependent manner on BV-2 cells and PC-12 cells.

### 2.7. Evaluation of Anti-Insulin Resistance and Anti-Cancer Activity of HM475 In Vitro

**Figure 5:**
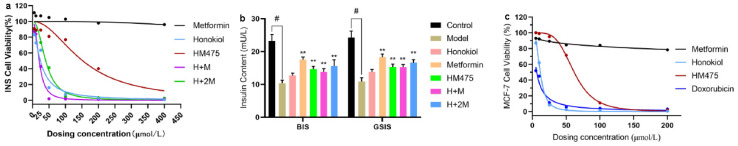
(**a**) Effect of administration group on the viability of INS-1 cell. (**b**) Influence of administration group on insulin of ins-1 cells stimulated by high glucose. BIS group: 5.6 mmoL/L glucose. GSIS group: 16.7 mmoL/L glucose. # *p* < 0.01, ** *p* < 0.01. (**c**) Effect of administration group on the viability of MCF-7 cell.

Figure 5a showed that honokiol was the most toxic to INS-1 cells, and the toxicity of HM475 and a mixture group (Honokiol + Metformin) to INS-1 cells was significantly lower than that of honokiol. The IC_50_ value for HM475 was 115.95 ± 6.32 μmoL/L, which is lower than that of metformin. In addition, there was a significant difference between the model group and the blank group (*p* < 0.01), indicating that the BIS (5.6 mmoL/L glucose broth) and GSIS (16.7 mmoL/L glucose broth) groups had been successfully modeled in the experiment. After metformin intervention, the insulin contents were 17.52 mU/L (BIS) and 18.25 mU/L (GSIS), which were much higher than those in the model group. The insulin contents in the HM475-treated group were 14.74 mU/L (BIS) and 15.22 mU/L (GSIS), implying that HM475 has an excellent anti-insulin resistance activity compared with model group (Figure 5b). An increasing number of studies show that honokiol has anti-cancer, anti-inflammatory, antibacterial, and antiviral effects. Therefore, the inhibitory effect of the derivative HM475 on the proliferation of human breast cancer cells MCF-7 in vitro was investigated. As shown in Figure 5c, the toxicity of HM475 to MCF-7 cells was greatly reduced compared with honokiol. Therefore, we can conclude that HM475 displays moderate inhibitory efforts (IC_50_ = 61.28 ± 2.11 μmoL/L) compared with those of Doxorubicin (IC_50_ = 6.09 ± 0.13 μmoL/L) and Honokiol (IC_50_ = 11.42 ± 0.14 μmoL/L).

## 3. Experimental Section

### 3.1. Synthetic Procedure for HM475

Quantities of 0.04 moL of honokiol, 0.08 moL of anhydrous Na_2_CO_3_ and 200 mL of anhydrous methanol were added into a clean three-necked flask, and the temperature was raised to 40 °C after stirring. In the stirring process, 16 mL of methyl bromide acetate (0.17 moL) was added, keeping the temperature below 50 °C. After dripping, the temperature rose to 65 °C. Reflux reaction was for 8 h. At the end of the reaction, the filtrate was filtered while hot, and the filtrate was steamed to obtain an oily, light yellow liquid (intermediate product HM410 (Scheme 1, **3a**). Metformin hydrochloride (0.04 moL), sodium methoxide-methanol solution (50 mL) and anhydrous methanol (20 mL) were stirred at room temperature for 30 min, then compound **3a** was slowly added into the above solution. The mixture was then refluxed at 65 °C for 12 h. With the progress of the reaction, the color of the solution finally changed from colorless to red. The reaction solution was vacuumed suction filtration. Then, the precipitate was collected and dissolved in NaOH solution (pH 10~12), then a solution of hydrochloric acid was added. Finally, the pale yellow precipitate (HM475) was obtained. Yield: 17.8% (3.471 g). ^1^H-NMR (500 MHz, DMSO-*d*_6_) δ 3.07 (6H, s, H-14, H-15), 3.31 (2H, d, *J* = 6.5 Hz, H-3′), 3.38 (2H, d, *J* = 7.0 Hz, H-3), 4.72 (2H, s, H-10′), 4.76 (2H, s, H-10), 4.97–5.06 (4H, m, H-1, H-1′), 5.93–5.99 (2H, m, H-2, H-2′), 6.91 (2H, d, *J* = 8.5 Hz, H-6, H-6′), 7.02 (1H, s, H-7′), 7.38 (1H, s, H-9) 7.49 (1H, s, H-9′), 7.06 (1H, s, H-5). ^13^C-NMR (125 MHz, DMSO-*d*_6_) 172.63 (C-11), 165.08 (C-11′), 166.55(C-13), 170.32 (C-12), 153.54 (C-7), 127.40(C, C-7′), 138.05 (C-2′), 136.89 (C-2), 132.96 (C-4, C-4′), 131.86 (C-9), 130.84(C-9′), 128.24 (C-5), 154.40(C-5′), 128.24 (C-8, C-8′), 112.12 (C-6),113.86(C-6′), 115.48 (C-1,C-1′), 70.45(C-10), 65.87(C-10′), 38.64 (C-3), 35.01 (C-3′), 35.64 (C-14, C-15).

### 3.2. Crystal Structure Determination and Refinement of HM475

X-ray diffraction measurements were performed on a Bruker Smart CCD area detector in the range 4.461 < θ < 73.51^o^ with CuKα radiation (λ = 1.5418 Å) at 100 K. All empirical absorption corrections were applied by using the SADABS program [32]. The structures were determined using pattern methods, which yielded the positions of all non-H atoms. All H atoms of the complexes were placed in calculated positions with fixed isotropic thermal parameters, and the structure factor calculations were included in the final stage of full-matrix least squares refinement. All calculations were performed using the SHELXTL-97 system of the computer programs [33].

### 3.3. Determination of Physical and Chemical Properties, Impurity Inspection and Content Determination

According to the Chinese Pharmacopoeia operation method Chinese Pharmacopoeia (2020) Volume 4, the physical and chemical properties of HM475 were inspected, and the color aggregation state, odor and taste of the product were observed; the solubility, hygroscopicity and melting point of the compound were measured. Impurities such as chloride, iron salt, loss on drying, residue on ignition, heavy metal inspection and arsenic salt were detected according to the Chinese Pharmacopoeia operation method Chinese Pharmacopoeia (2020) Volume 4, which involved precisely weighing 200.0021 mg of HM475, which was dissolved in anhydrous methanol and diluted to a volume of 100 mL in a volumetric flask to prepare a solution of 200 μg/mL HM475. Then, strictly following the requirements of Chinese Pharmacopoeia (2020) Volume 4 to carry out relevant content determination experiments.

Chromatographic analyses were performed on a 2695 separation module coupled to a 2487 dual λ absorbance detector assisted by Empower software (Waters, Milford, MA, USA). HM475 was dissolved in anhydrous methanol to make 600 μg/mL sample solution. Five calibration standards of HM475 were prepared in the concentration range from 60~400 µg/mL by diluting the stock solution in anhydrous methanol. The injection volume was 10 µL. Samples were injected onto a C18 column (JADE-PAK ODS-AQ, 250 × 4.6 mm, 5 µm, TechWay, Shanghai, China) heated to 30 °C. The analytes were separated by an isocratic elution with acetonitrile (A) and 0.2% phosphoric acid water (B) at a flow rate of 1 mL/min 30 min (A:B = 55%:45%). The 266 nm detection wavelength was monitored.

### 3.4. Cell Culture and Determination of Cell Viability

RAW264.7 cells were kindly provided by the New Drug Research and Development Center of Guangdong Pharmaceutical University. Rat insulin cells INS-1 and Human breast cancer cells MCF-7 were purchased from Saiqi Shanghai Biology Company (Shanghai, China); cultured in Dulbecco’s modified Eagle medium (DMEM), 10% fetal bovine serum (FBS, Gibco, Thermo Fisher, Shanghai, China) and 1% penicillin-streptomycin (Gibco) were heated in water bath at 37 °C before used), shaking the cryovial constantly for 1–2 min to completely thaw. Following the suppliers instructions to cell culture (at 37 °C, in an atmosphere of 5% CO_2_). This culture condition also applies for the cell lines experiments. The cell viability was determined by MTT. Cells (1 × 10^4^ cells/mL) were plated in 96-well plates and allowed to attach for 24 h at 37 °C in an atmosphere with 5% CO_2_. Then, 100 μL of MTT-containing medium was added per well (5 mg/mL MTT stock solution + 80 microliters of blank medium) and incubated for 4 h. Finally, 150 µL of DMSO was added to each well, including shaking and mixing, and the absorbance was measured at 490 nm.

### 3.5. Evaluation of Anti-Inflammatory, Insulin Resistance (IR), Neuroprotective and Anti-Breast Cancer Activities

Adjusting the cell density to 10^4^ cells/mL, it was inoculated in a 96-well plate, 100 µL per well, and incubated in a 37 °C, 5% CO_2_ incubator for 24 h. The supernatant was carefully aspirated and the appropriate drug concentration was selected according to the results of the cell culture experiment. After 1 h of administration, an appropriate amount of Lipopolysaccharide (LPS) was added to the inflammation model group and the administration group. To make the final concentration 0.5 µg/mL, the blank group was given the same amount of PBS, the culture was continued for 48 h, and the supernatant was collected. The kit instructions determined the content of COX-2 and PGE2, and the Griess method was used to detect the content of NO. Ibuprofen [14] remained as the positive group throughout the anti-inflammatory experiment. The operation is as before: 50 µL of the cell supernatant of each administration group were taken and the standard dilution of each concentration in a 96-well plate, 50 µL of solution A was added, and then 50 µL of solution B was added, mixed well, and the absorbance was measured at 450 nm within 2 min. The ELISA Calc software was used for the calculation, the standard curve was drawn by linear regression, and the curve equation was calculated. The cells were taken in the logarithmic growth phase, digested and counted, and the cell concentration was adjusted to 1 × 10^4^ cells/mL, inoculated in 96-well plates, and after 24 h of pre-culture, the supernatant culture medium was carefully aspirated and added to the other groups, except for the blank group. Medicated medium containing 40 mmoL/L glucose, intervened for 48 h for modeling, was carefully aspirated and the supernatant was discarded. We added 100 μL of sugar-free medium, equilibrated for 30 min, and each group was given 5.6 mmoL/L (BIS) or 16.7 mmoL/L (GSIS) glucose culture medium, 100 μL/well, cultured at 37 °C for 1 h. We took the supernatant, stored it at −20 °C, and measured the amount of insulin secretion in each group according to the ELISA kit. Metformin also acted as a positive drug in IR research. The operation methods of PC-12, BV-2 and MCF-7 cell experiments are similar to RAW264.7 cell experiment.

### 3.6. General Apparatus and Chemicals

SZCL-2 digital display intelligent temperature control magnetic stirrer (Yuhua Instrument Co., Ltd., Gongyi, China); BP210D analytical balance (Sartorius); Nuclear magnetic resonance (NMR) Avance III-500 (Bruker, Bremen, Germany); 2487/2695 high performance liquid chromatograph (Waters, USA); C18 column (JADE-PAK ODS-AQ, 250 × 4.6 mm, 5 µm) (TechWay, China); rotary Evaporator (EYELA Company, Tokyo, Japan); DU-800 ultraviolet-visible spectrophotometer (BECKMAN COULTER, Tokyo, Japan); Fourier infrared spectrometer (Bruker VECTOR22FT-IR infrared spectrometer); SZCL-3A temperature-controlled magnetic stirrer (Korui Instrument Co., Ltd., Shantou, China); Kafler micro melting point tester (Beijing Taike Instrument Co., Ltd., Beijing, China); UPLC-ESI-Q/TOF-MS, MassLynx4.1 data processing system; ACQUITY UPLC TM BEH C18 column (2.1 × 50 mm, 1.7 μm) (Waters, USA).

Honokiol (Hunan Heguang Biotechnology Co., Ltd., Changsha, China). Metformin hydrochloride (Wuhan Yuancheng Co-creation Technology Co., Ltd., Wuhan, China). Sigma Company (Cream Ridge, NJ, USA) provided lipopolysaccharide (LPS) and ibuprofen. Fetal bovine serum (FBS), Dulbecco’s modified eagle medium (DMEM), 0.25% trypsin and penicillin-streptomycin double antibody were from Gibco Company. Acetonitrile and methanol (HPLC grade) come from Merk, Methyl bromoacetate from Mackllin (Shanghai, China).

## 4. Conclusions

All the activity experiments showed that the cyto-toxicity of the derivative HM475 was significantly reduced after structural modification compared to honokiol. The results of the anti-inflammatory experiment showed that HM475 has anti-inflammatory activity, and the inhibitory ability of HM475 on the iNOS enzyme and COX-2 enzyme was much higher than that of the positive drug ibuprofen and the raw material drug honokiol. Its anti-inflammatory effects mainly inhibited serial COX, reduced inflammatory cytokines, activated various downstream mediators, and blocked the NF-κB pathway. In terms of neuroprotection, HM475 exhibited favorable inhibition of the inflammatory factors NO, TNF-α and IL-1βp. The anti-IR experiment showed that insulin levels increased after HM475 intervention, and there were significant differences with the model group, indicating that HM475 could alleviate the insulin resistance of INS-1 cells stimulated by high glucose. Anticancer experiments showed that the anticancer activity was also reduced due to the reduced toxicity of the derivative, but HM475 still showed a certain activity with its IC_50_ value of 61.28 ± 2.11 μmoL/L. The anti-tumor mechanism of HM475 may be similar to that of honokiol, activating related apoptosis proteins such as related caspase-3, caspase-9 and Bcl-2, thereby inducing caspase multi-pathway apoptosis, and inhibiting tumor cells and inducing its apoptosis through signal transduction pathways such as MEK, p38-MAPK and JNK signaling pathways. The above results show that by the cyclization of metformin and honokiol to form a new binuclear molecule, the toxicity is reduced while the activities of anti-inflammatory and insulin resistance are unchanged, and even rising. In this study, guided by the theory of traditional Chinese medicine through the principle of molecular splicing, metformin and honokiol were first combined through methyl bromoacetate to form a new derivative, HM475. In addition, the quality control of HM475 was constructed for the first time.

## Figures and Tables

**Table 1:** Table Screening of Reaction Conditions.

Entry	Base (Equiv)	Solvent	Temp (°C)	Yield (%)
1	Na_2_CO_3_ (2.0)	MeOH	65	77.5
2	Na_2_CO_3_ (2.0)	EtOH	65	24.1
3	Na_2_CO_3_ (2.0)	MeCN	65	22.9
4	K_2_CO_3_ (2.0)	MeOH	65	18.9
5	K_2_CO_3_ (2.0)	EtOH	65	5.9
6	K_2_CO_3_ (2.0)	MeCN	65	5.6
7	Na_2_CO_3_ (2.0)	MeOH	25	37.5
8	Na_2_CO_3_ (2.0)	MeOH	45	47.9
9	Na_2_CO_3_ (2.0)	MeOH	67	69.3
10	Na_2_CO_3_ (1.5)	MeOH	65	63.4
11	Na_2_CO_3_ (1.0)	MeOH	65	42.1

Reaction conditions: **1a** (0.04 moL), **2a** (0.08 mmoL), base, solvent (200 mL), 8 h.

**Table 2:** Screening Reaction Conditions of honokiol derivatives (**1**) and metformin (**2**).

Entry	Base	Solvent	Temp (°C)	Yield (%)
1	Na_2_CO_3_	MeOH	65	10.8
2	Na_2_CO_3_	EtOH	65	7.4
3	Na_2_CO_3_	MeCN	65	4.9
4	K_2_CO_3_	MeOH	65	14.3
5	K_2_CO_3_	EtOH	65	9.8
6	K_2_CO_3_	MeCN	65	6.6
7	NaOH	MeOH	65	7.3
8	NaOH	EtOH	65	5.0
9	NaOH	MeCN	65	3.3
10	MeONa	EtOH	65	12.2
11	MeONa	DMF	65	1.2
12	MeONa	MeCN	65	8.2
13	MeONa	MeOH	25	11.1
14	MeONa	MeOH	45	11.5
15	MeONa	MeOH	65	17.8
16	MeONa	MeOH	67	16.8

Reaction conditions: **1** (0.04 moL), **2** (0.08 mmoL), base, solvent (50 mL), 8 h.

**Table 3:** Crystal data for HM475.

Empirical Formula	C_26_H_29_N_5_O_4_
Mr	476.55
T [K]	100
Crystal system	Triclinic
Space group	P-1
a [Å]	4.2879(3)
b [Å]	15.4405(9)
c [Å]	19.29(12)
α [°]	107.806(6)
β [°]	96.102(6)
γ [°]	95.991(6)
V [Å^3^]	1196.61(14)
Z	2
D_c_ [gcm^–3^]	1.323
θ range for data collection [°]	4.461–73.512
Limiting indices, hkl	−5 ≤ h ≤ 3; −17 ≤ k ≤ 19; −23 ≤ l ≤ 23
Reflections collected	4656
Independent reflections (R_int_)	3616
Good-of-fit on *F*^2^	0.996
*R*_1_/*wR*_2_ [*I* > 2σ(*I*)] ^[a]^	0.0875/0.0683

^[a]^ *R*_1_ = Σ||*F*_o_| – |*F*_c_||/Σ|*F*_o_|, *wR*_2_ = [Σ*w*(*F*_o_^2^ – *F*_c_^2^)^2^/Σ*w*(*F*_o_^2^)^2^]^1/2^.

## Data Availability

Not applicable.

## Supplementary Materials

The following supporting information can be downloaded at: https://www.mdpi.com/article/10.3390/molecules28093753/s1, Figure S1. UV-Visspectrum of HM475. Figure S2. Infrared (IR) spectrum of HM475. Figure S3. 1H NMR and 13C NMR (DMSO-*d*_6_) spectrum of HM475. Figure S4. 1H-1HCOSY spectrum of HM475. Figure S5. HMBC spectrum of HM475. Figure S6. Dept spectrum of HM475. Figure S7. QC spectrum of HM475. Figure S8. HRMS spectrum of HM475. Figure S9. HM475 HPLC chromatogram. Figure S10. Ortep diagram of HM475. Figure S11. Data of the Ortep diagram; Table S1 ^1^H and ^13^C NMR data for HM475 (DMSO-*d*_6_). Table S2 Solubility of HM475. Table S3 Appearance and smell of HM475. Table S4 Results of moisture absorption determination of HM475. Table S5 Results of melting point determination of HM475. Table S6 Results of chloride inspection. Table S7 Results of iron salt inspection. Table S8 Results of determination of weight loss by drying. Table S9 Results of determination of burning residue. Table S10 Results of inspection of heavy metal lead. Table S11 Results of arsenic salt inspection. Table S12 Results of precision of instrument. Table S13 Results of intra-day precision. Table S14 Results of daytime precision. Table S15 Results of repetitive test results. Table S16 Results of recovery test results. Table S17 The influence of various factors on HM475. Table S18 Results of accelerated test. Table S19 Results of long-term test.

## Sample Availability Statement

Not available.
