# Supplementary material for: Synthesis, Quality Control and Preliminary Activity Evaluation of a New Compound HM475
Source: Molecules. 2023 Apr 27;28(9):3753. doi: 10.3390/molecules28093753 (PMC10180444; doi:10.3390/molecules28093753)
Supplement: Supplementary file 1 [file molecules-28-03753-s001.zip › molecules-2329911-supplementary.pdf]

Synthesis, Quality Control And Preliminary Activity Evaluation of a New Compound HM475

Jieqing Guo [1,2], Luming Xie [1,2], Jing Zhang 1,2, Han Cao [1,2], Juanxia Wang [1,2], Xia Wu [1,2,*] and Yifan Feng [1,2,*]

[1] New Drug Research and Development Center, Guangdong Pharmaceutical University, Guangzhou 510006, China

[2] Guangdong Provincial Key Laboratory of Advanced Drug Delivery, Guangdong Provincial Engineering Center of Topical Precise Drug Delivery System, Guangdong Pharmaceutical University, Guangzhou 510006, China

* Correspondence: wxiaxia@163.com (X.W.); yffeng@gdpu.edu.cn (Y.F.)

Supplementary Information

Figure S1 UV-Vis spectrum of HM475.

Figure S2 Infrared (IR) spectrum of HM475.

Figure S3 ^{1}H NMR and ^{13}C NMR (DMSO-d_6) spectrum of HM475.

Figure S4 ^{1}H-^{1}HCOSY spectrum of HM475.

Figure S5 HMBC spectrum of HM475.

Figure S6 Dept spectrum of HM475.

Figure S7 QC spectrum of HM475.

Figure S8 HRMS spectrum of HM475.

Figure S9 HM475 HPLC chromatogram.

Figure S10 Ortep diagram of HM475.

Figure S11 Data of the Ortep diagram.

Table S1 ^{1}H and ^{13}C NMR data for HM475 (DMSO-d_6).

Table S2 Solubility of HM475.

Table S3 Appearance and smell of HM475.

Table S4 Results of moisture absorption determination of HM475.

Table S5 Results of melting point determination of HM475.

Table S6 Results of chloride inspection.

Table S7 Results of iron salt inspection.

Table S8 Results of determination of weight loss by drying.

Table S9 Results of determination of burning residue.

Table S10 Results of inspection of heavy metal lead.

Table S11 Results of arsenic salt inspection.

Table S12 Results of precision of instrument.

Table S13 Results of intra-day precision.

Table S14 Results of daytime precision.

Table S15 Results of repetitive test results.

Table S16 Results of recovery test results.

Table S17 The influence of various factors on HM475.

Table S18 Results of accelerated test.

Table S19 Results of long-term test.

Figure S1 UV spectrum of HM475

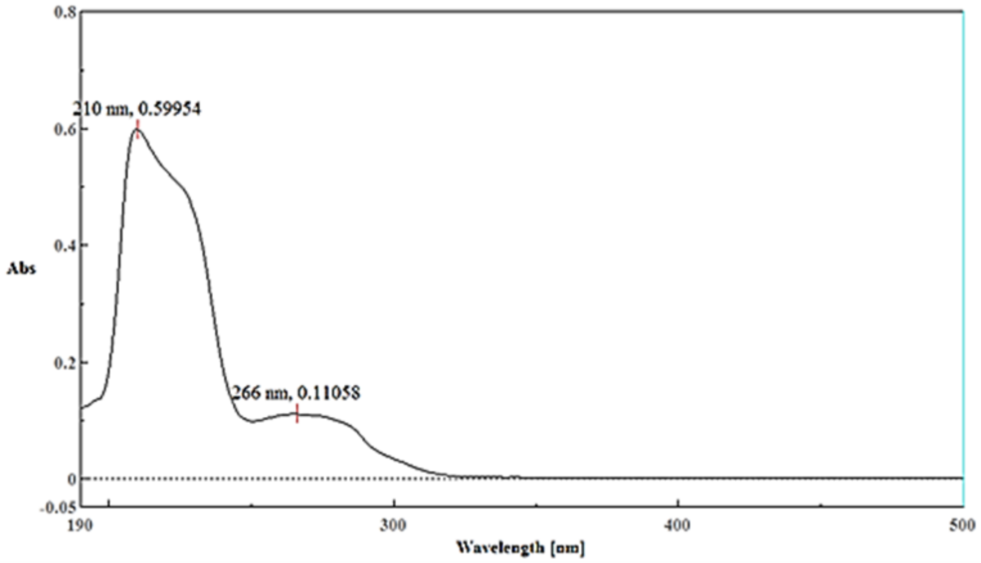

Figure S2 Infrared (IR) spectrum of HM475

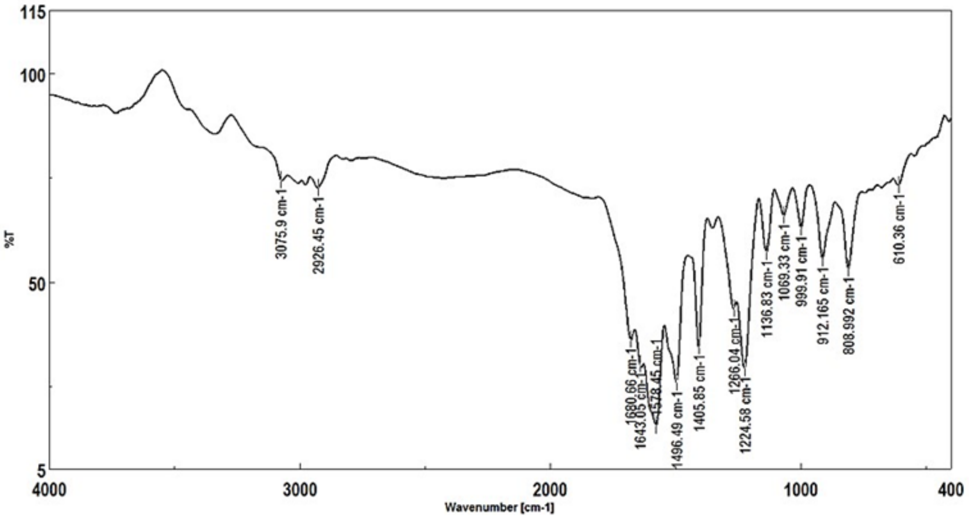

Figure S3 ^{1}H NMR and ^{13}C NMR (DMSO-d6) spectrum of HM475.

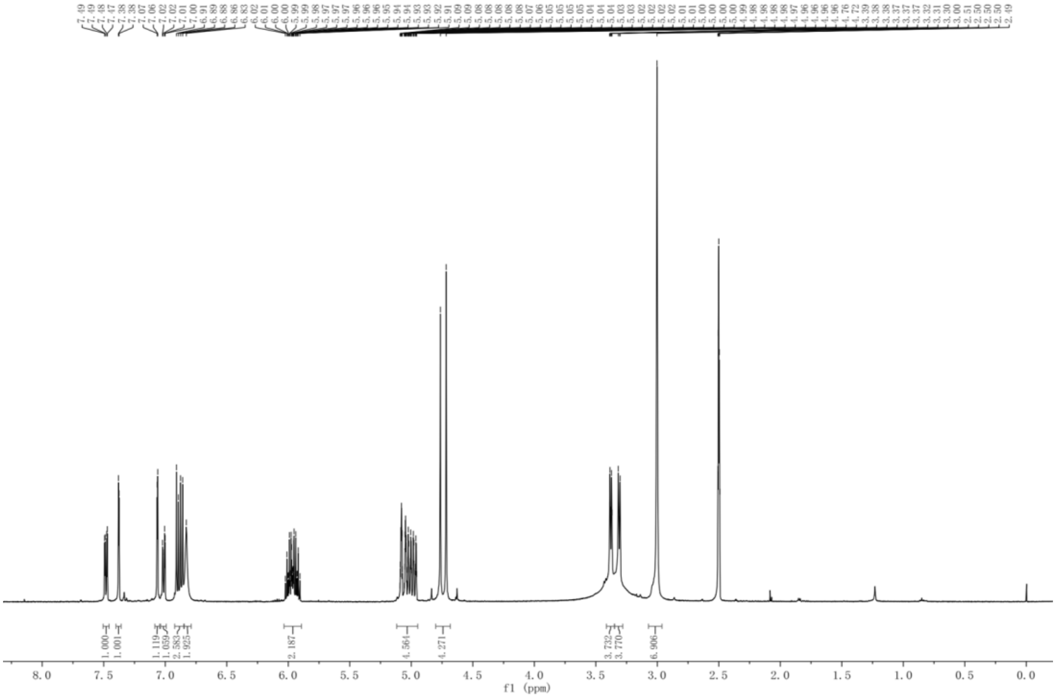

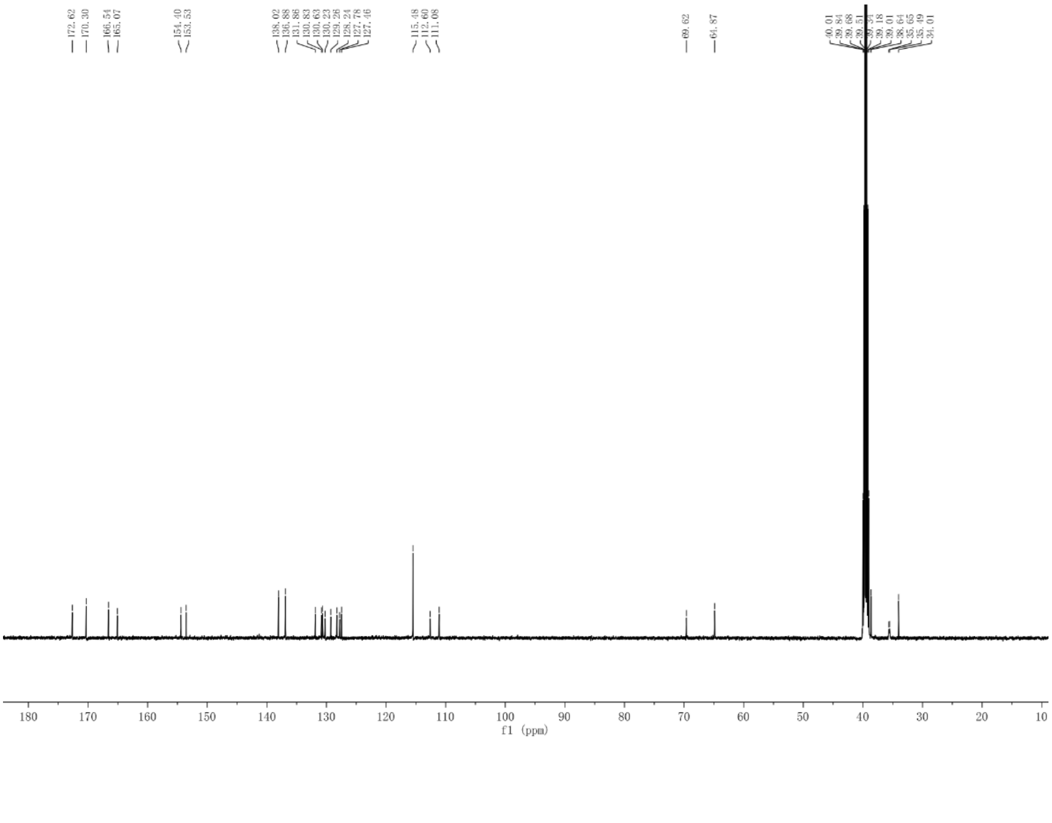

Figure S4 ^{1}H-^{1}HCOSY spectrum of HM475

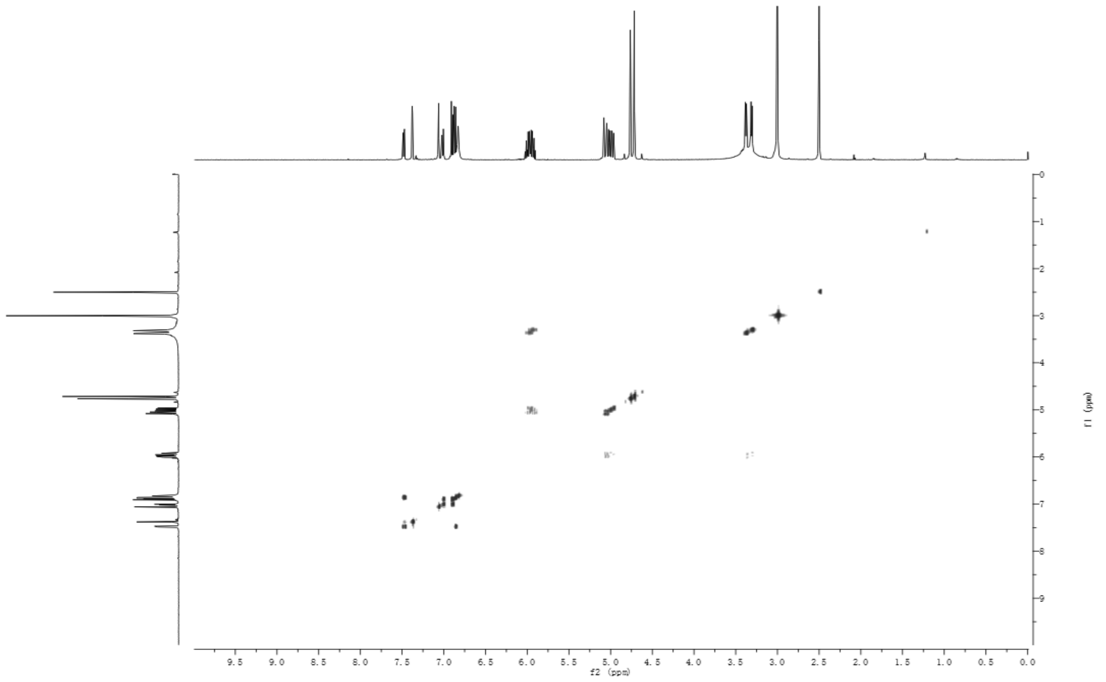

Figure S5 HMBC spectrum of HM475

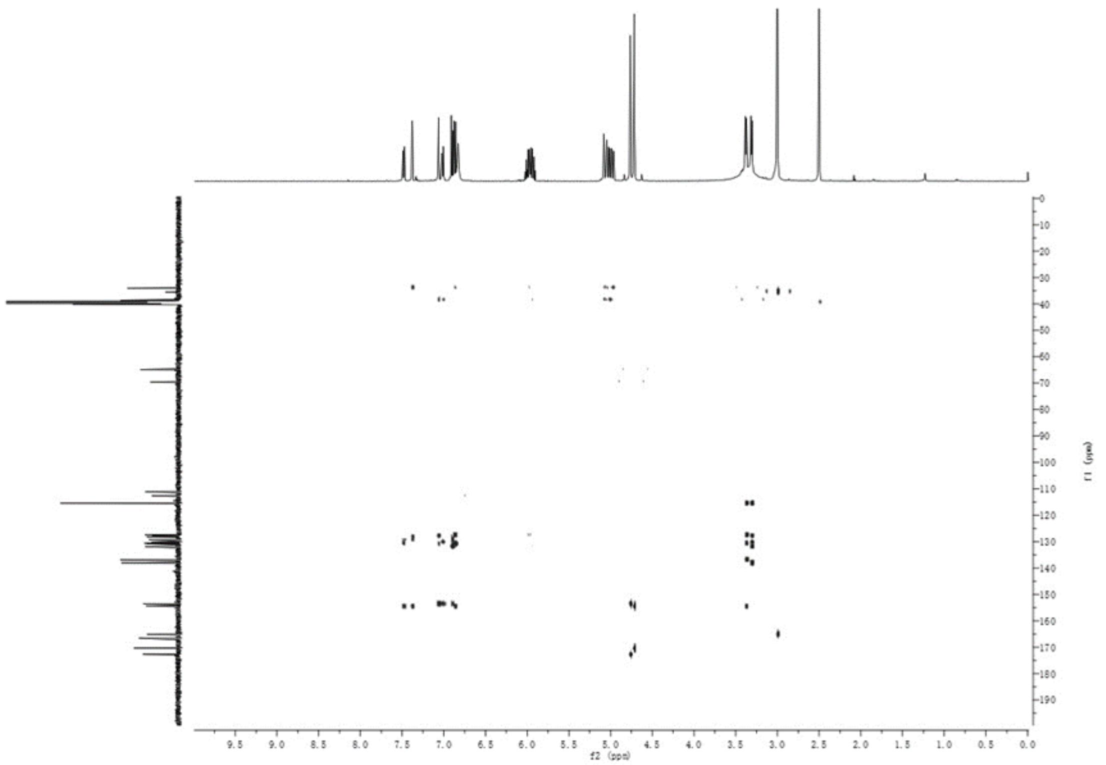

Figure S6 Dept-135°spectrum of HM475

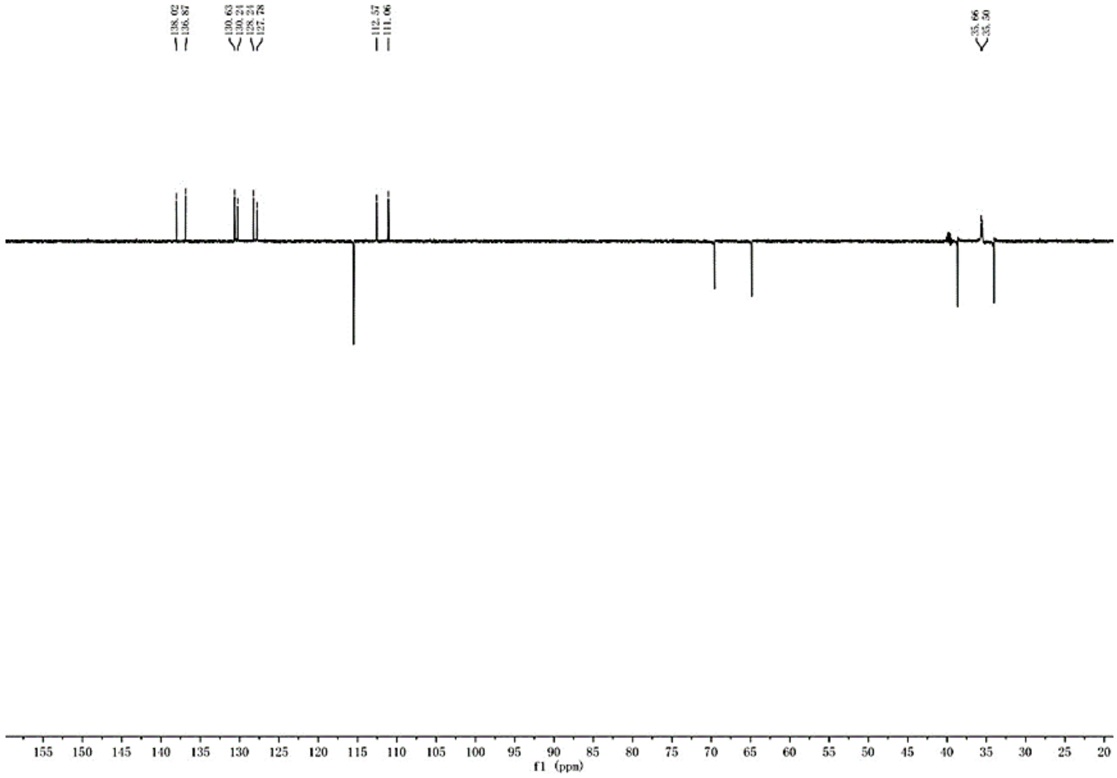

Figure S7 QC spectrum of HM475

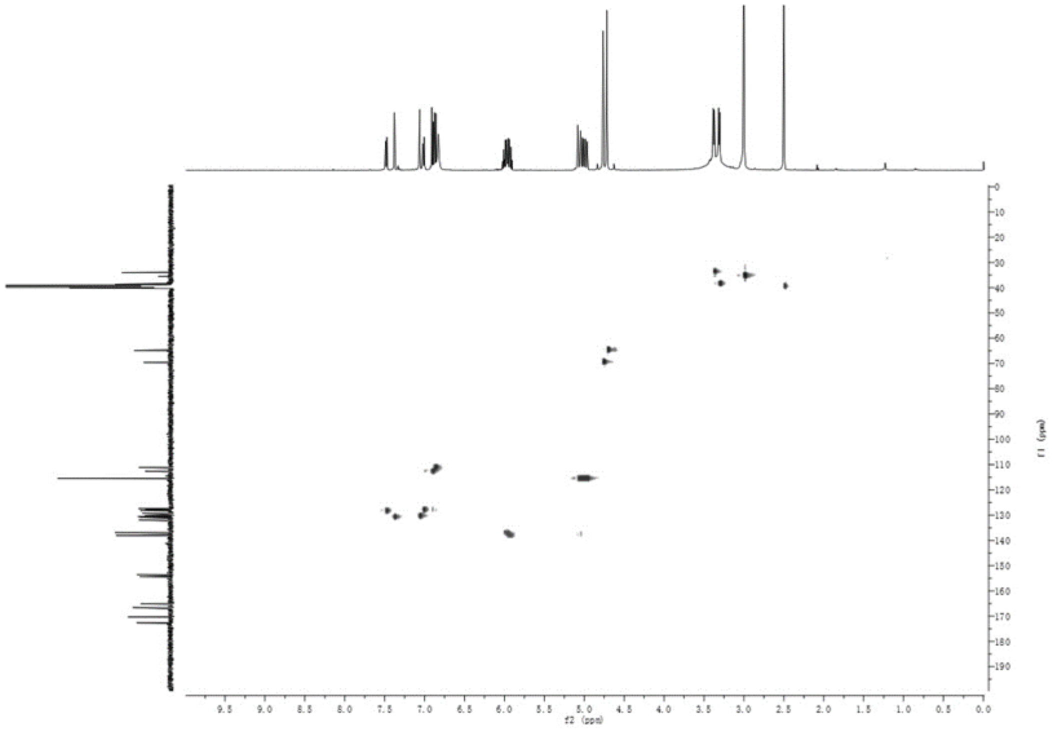

Figure S8 HRMS spectrum of HM475

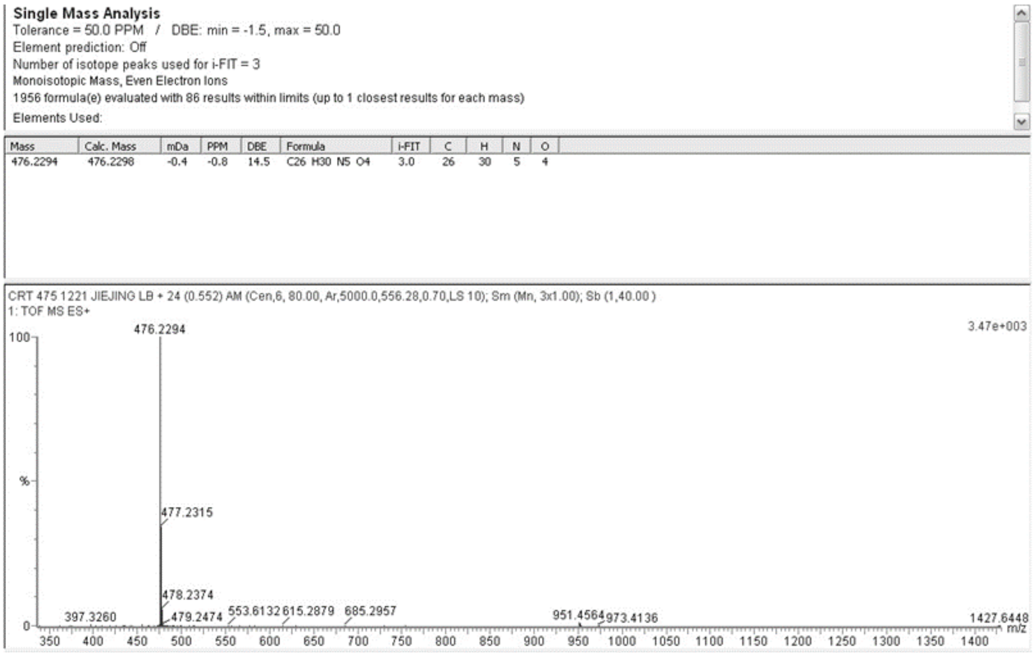

Figure S9 HM475 HPLC Chromatogram (266nm).

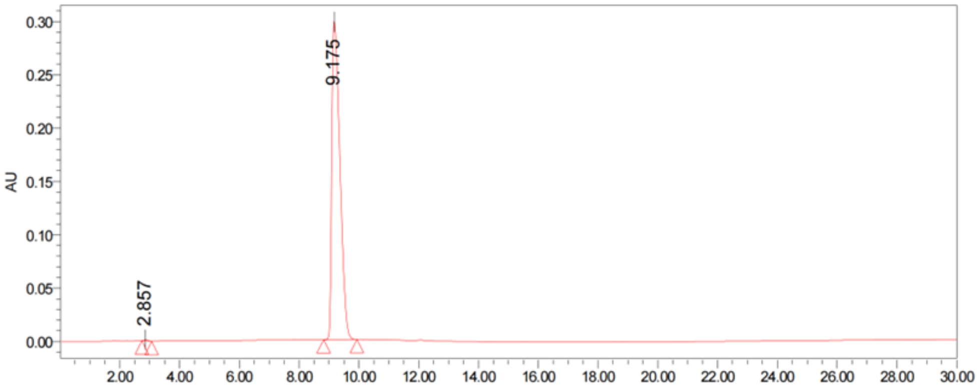

Figure S10 Ortep diagram of HM475.

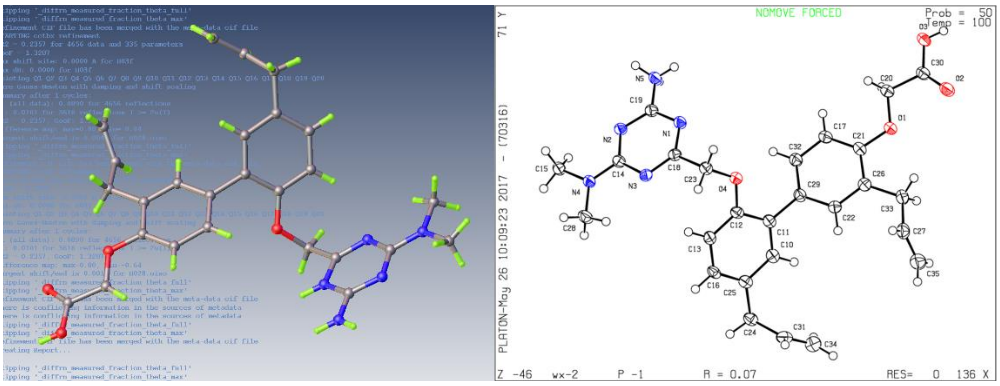

Figure S11 Data of the Ortep diagram

```
Bond precision:   C-C = 0.0042 A          Wavelength=1.54178

Cell:           a=4.2879(3)        b=15.4405(9)     c=19.2940(12)
                alpha=107.806(6)   beta=96.102(6)   gamma=95.991(6)
Temperature:    100 K

                Calculated                  Reported
Volume          1196.62(14)                 1196.61(14)
Space group     P -1                        P -1
Hall group      -P 1                        -P 1
Moiety formula  C26 H30 N5 O4               C26 H30 N5 O4
Sum formula     C26 H30 N5 O4               C26 H30 N5 O4
Mr              476.55                      476.55
Dx,g cm-3       1.323                       1.323
Z               2                           2
Mu (mm-1)       0.742                       0.742
F000            506.0                       506.0
F000'           507.54
h,k,lmax        5,19,23                     5,19,23
Nref            4838                        4656
Tmin,Tmax       0.762,0.849                 0.669,1.000
Tmin'           0.682

Correction method= # Reported T Limits: Tmin=0.669 Tmax=1.000
AbsCorr = MULTI-SCAN

Data completeness= 0.962          Theta(max)= 73.512

R(reflections)= 0.0682( 3616)     wR2(reflections)= 0.2276( 4656)

S = 1.225                    Npar= 320
```

Table S1 ^{1}H and ^{13}C NMR data for HM475 (DMSO-d_6)

Location	HM475	
	δH	δC
1，1’	5.02 (4H, m)	115.48
2	5.93(1H, m)	136.89
2’	5.99(1H, m)	138.05
3	3.38 (2H, d, *J* = 7.0 Hz)	38.64
3’	3.31 (2H, d, *J* = 6.5 Hz)	35.01
4		132.96
4’		132.96
5	7.06 (1H, s)	128.24
5’		154.40
6	6.91 (1H, d，*J* = 8.5 Hz))	112.12
6’	6.91(1H, d, *J* = 8.5 Hz)	113.86
7		153.54
7’	7.02 (1H, s)	127.40

8		128.24
8’		128.24
9	7.38 (1H, s)	130.84
9’	7.49(1H,S)	130.84
10	4.76 (2H, s)	70.45
10’	4.72 (2H, s)	65.87
11		172.63
11’		165.08
12		170.32
13		166.55
14，15	3.07(6H, s)	35.64

、

Table S2 Solubility of HM475

Solvents	Sample weight(mg)	Add solvent volume (mL)	Solvent volume per gram of sample (mL)	Dissolution	Conclusion
Methanol	10	0.01	1	insoluble	Very slightly soluble
		0.1	10	insoluble	
		0.3	30	insoluble	
		1.0	100	insoluble	
		3.0	300	insoluble	
		10	1000	insoluble	
		90	9000	dissolve	
0.1mol/L NaOH	10	0.01	1	insoluble	Dissolve
		0.1	20	insoluble	
		0.2	20	dissolve	
Ethanol	10	0.01	1	insoluble	Almost insoluble
		0.1	10	insoluble	
		0.3	30	insoluble	
		1.0	100	insoluble	
		3.0	300	insoluble	
		10	1000	insoluble	

		100	10000	insoluble	
0.1mol/L HCl	10	0.01	1	insoluble	Almost insoluble
		0.1	10	insoluble	
		0.3	30	insoluble	
		1.0	100	insoluble	
		3.0	300	insoluble	
		10	1000	insoluble	
		100	10000	not completely dissolved	
Water	10	0.01	1	insoluble	insoluble
		0.1	10	insoluble	
		0.3	30	insoluble	
		1.0	100	insoluble	
		3.0	300	insoluble	
		10	1000	insoluble	
		100	10000	insoluble	

Table S3 Appearance and smell of HM475

Batch	color	appearance	smell
190304	light yellow	powder	Odorless and tasteless
190313	light yellow	powder	Odorless and tasteless
190402	light yellow	powder	Odorless and tasteless

Table S4 Results of moisture absorption determination of HM475

Batch	A(g)	B(g)	C(g)	D(g)	hygroscopicity (%)
190304	26.5683	27.7792	27.7828	0.0036	0.30
190313	26.3772	27.4936	27.4965	0.0029	0.26
190402	25.3546	26.5669	26.5702	0.0033	0.27

A: Weighing bottle B: Sample + weighing bottle weight

C: Sample + weighing bottle weight after 24 days D: Gain weight

Table S5 Results of melting point determination of HM475

Batch	Initial melting temperature (℃)	full melting temperature (℃)
190304	169	172
190313	170	171
190402	170	172

Table S6 Results of chloride inspection

Batch	Standard Sodium Chloride Solution			
	0.5 ml	1.0 ml	2.0 ml	5.0 ml
190304	+	-	-	-
190313	+	+	-	-
190402	+	-	-	-
190415	+	-	-	-

Table S7 Results of iron salt inspection

Batch	Standard iron solution		
	0.5 ml	1.0 ml	2.0 ml
190304	-	-	-

190313	-	-	-
190402	o	-	-
190415	-	-	-

Table S8 Results of determination of weight loss by drying

Batch	sample weight (g)	Dehumidifying weight (g)	Loss on drying
190304	1.0001	0.0018	0.18%
190313	1.0001	0.0022	0.22%
190402	0.9999	0.0016	0.16%
190415	1.0003	0.0025	0.25%

Table S9 Results of determination of burning residue

Batch	sample weight (g)	Residue weight (g)	Residue content
190304	2.0036	0.0010	0.10%
190313	2.0054	0.0013	0.13%
190402	2.0058	0.0009	0.09%
190415	2.0060	0.0011	0.11%

Table S10 Results of inspection of heavy metal lead

Batch	Standard lead solution			
	0.5 ml	1.0 ml	1.5 ml	2.0 ml
190304	-	-	-	-
190313	o	-	-	-
190402	-	-	-	-

190415	-	-	-	-

Table S11 Results of arsenic salt inspection

Batch	testing sample 0.5 g	testing sample 1.0 g	Testing sample 2.0 g
190304	-	-	-
190313	-	-	-
190402	-	-	-
190415	-	-	-

Table S12 Results of precision of instrument

Injections number	Peak A	Average	RSD(%)
1	5229548		
2	5243656		
3	5222918		
4	5233880	5230368.3	0.16
5	5232531		
6	5219677		

Table S13 Results of intraday precision

Injection time (h)	80%	100%	120%
0	3963004	5287325	5776148

2	3955404	5295198	5768669
4	3936798	5266095	5723918
6	3940639	5271426	5731581
8	3940294	5276209	5742899
10	3950692	5287331	5748657
12	3988241	5306074	5759117
Average	3953581.7	5284236.9	5750141.3
RSD%	0.45	0.26	0.33

Table S14 Results of daytime precision

Injection time（day）	80%	100%	120%
1	3963004	5287325	5776148
2	3955404	5295198	5768669
3	3936798	5266095	5723918
Average	3980951	5309795	5788250.3
RSD%	1.01	0.48	0.41

Table S15 Results of repetitive test results

Number	Peak area	Average	RSD（%）
1	5221365		

2	5214517		
3	5199627		
4	5195805	5205502.18	0.16
5	5203369		
6	5198330		

Table S16 Results of recovery test results

Serial number	Weight （g）	Peak area	Measured （g）	Recovery （%）	Average （%）	RSD （%）
80%-1	0.02005	3960242	0.02007	100.13		
80%-2	0.02006	3946778	0.02001	99.74	100.05	0.23
80%-3	0.02003	3962294	0.02009	100.28		
100%-1	0.02503	5212628	0.02500	99.88		
100%-2	0.02504	5198600	0.02493	99.57	99.82	0.18
100%-3	0.02506	5225353	0.02506	100.00		
120%-1	0.03001	5753869	0.02992	99.67		
120%-2	0.03003	5771713	0.03001	99.93	99.87	0.14
120%-3	0.03004	5776815	0.03004	100.00		

Table S17 The influence of various factors on HM475

HM475 content（%）	high temperature	high humidity	direct sunlight
Zero day	99.16	99.16	99.16
Fifth day	98.96	98.66	98.88
Tenth day	98.57	98.21	98.76

Table S18 Results of accelerated test

HM475 content（%）	190304	190313	190402
Zero month	99.16	99.14	99.17
First month	99.01	98.99	99.11
Second month	98.83	98.79	98.89
Third month	98.65	98.69	98.56
Sixth month	98.27	98.16	98.23

Table S19 Results of long-term test

HM475 content（%）	190304	190313	190402
Zero month	99.16	99.14	99.17
Third month	99.05	98.93	99.08
Sixth month	98.83	98.87	98.81
Ninth month	98.69	98.52	98.62
Twelfth month	98.45	98.34	98.41
